# Nanoparticles alleviate non-alcoholic steatohepatitis via ER stress sensor-mediated intestinal barrier damage and gut dysbiosis

**DOI:** 10.3389/fmicb.2023.1271835

**Published:** 2024-03-07

**Authors:** Manman Zhu, Yong Cheng, Yue Tang, Shuojiao Li, Peng Rao, Guiyang Zhang, Lei Xiao, Jiatao Liu

**Affiliations:** ^1^Department of Pharmacy, The First Affiliated Hospital of Anhui Medical University, Hefei, Anhui, China; ^2^School of Pharmacy, Anhui Medical University, Hefei, Anhui, China; ^3^Department of Pharmacy, Anhui University of Chinese Medicine, Hefei, Anhui, China; ^4^Department of Pharmacology, School of Basic Medical Sciences, Anhui Medical University, Hefei, Anhui, China

**Keywords:** non-alcoholic steatohepatitis, endoplasmic reticulum stress, X-box binding protein 1, gut microbiota, lipopolysaccharide

## Abstract

**Introduction:**

The gut microbiota plays an important role in the development of non-alcoholic steatohepatitis (NASH), but the underlying mechanism is unclear. It has been found that the transcription factor XBP1s plays an important role in regulating inflammation and lipid metabolism and maintaining the integrity of intestinal barrier. However, whether XBP1s modulates the development of NASH by regulating the integrity of the intestinal barrier and altering the composition of the gut microbiota remains unknown.

**Methods:**

Mice fed with a fat-, fructose-, cholesterol-rich (FFC) diet for 24 weeks successfully established the NASH model, as demonstrated by significant hepatic steatosis, inflammation, hepatocyte injury and fibrosis. The profile of gut microbiota dynamically changed with the different stages of NAFLD via 16S rDNA sequencing the feces from mice fed with FFC diet for 0, 12, or 24 weeks or NASH mice treated with siRNA-loaded folic acid-modified TPGS (hereafter named FT@XBP1).

**Results:**

NASH mice had significantly higher abundance of Firmicutes, Blautia and Bacteroides, and lower abundance of Bifidobacterium and GCA-900066575. FT@XBP1 supplementation had a significantly attenuated effect on FFC diet-induced weight gain, hepatic fat accumulation, dyslipidemia, inflammatory cytokines, ER stress and fibrosis. In particularly, FT@XBP1 modulates the composition of the intestinal flora; for example, NASH mice demonstrated higher abundance of Blautia and Bacteroides, and lower abundance of Actinobacteriota, Muribaculaceae and Bifidobacterium, which were partially restored by FT@XBP1 treatment. Mechanistically, FT@XBP1 increased the expression of ZO-1 in the intestine and had the potential to restore intestinal barrier integrity and improve antimicrobial defense to alleviate enterogenic endotoxemia and activation of inflammatory signaling pathways.

**Discussion:**

Regulation of the key transcription factor XBP1s can partially restore the intestinal microbiota structure, maintain the integrity of intestinal mucosal barrier, and prevent the progression of NASH, providing new evidence for treating NASH.

## Background

The incidence of non-alcoholic fatty liver disease (NAFLD) is increasing year by year, with a global prevalence of ~25.24%, and it has become an important public health problem worldwide (Friedman et al., 2018; Younossi et al., 2018). NAFLD consists of a series of liver diseases, including non-alcoholic simple fatty liver (NAFL), non-alcoholic steatohepatitis (NASH) and NASH-associated cirrhosis. Approximately 25% of patients with NAFL can progress to NASH, which in turn may further develop into fibrosis, cirrhosis, and ultimately hepatocellular carcinoma (Huang et al., 2021). However, the precise mechanism of NAFL progression to NASH is still not fully understood, and there are no effective therapeutic interventions in the clinic. Therefore, in-depth study of the molecular mechanism of NAFL progression to NASH and an exploration of potential therapeutic targets are of great theoretical and practical value.

In recent years, the role of intestinal flora in the development of NAFL/NASH has received more and more attention. Early studies have found that intestinal flora can release some harmful substances to promote the progression of NAFL to NASH. For example, Gram-negative bacteria in humans can release lipopolysaccharide (LPS), which activates Kupffer cells and exacerbates inflammation in the liver (Seki et al., 2001). Clinical studies have found a significant positive correlation between endotoxemia and the severity of pathological lesions in NAFL, suggesting that LPS may play a crucial role in the transformation of NAFL into NASH (Vespasiani-Gentilucci et al., 2018). Carvalho et al. (2012) found that inhibition of bacterial overgrowth significantly reduced circulating levels of LPS and improved the insulin signaling and glucose tolerance in high-fat-fed mice, suggesting that increased intestinal permeability and elevated circulating LPS levels may play an indispensable role in the progression of NAFLD. In addition, one study found that the abundance of intestinal ethanol-producing bacteria was significantly increased in patients with NASH, along with significantly higher plasma levels of ethanol (Zhu et al., 2013). *In vivo* and *in vitro* studies have also found that endogenous ethanol not only promotes hepatocellular triglyceride deposition, but also promotes the production of reactive oxygen radicals and liver inflammation (Wang et al., 2021). The above results suggest that gut flora plays a vital role in the onset and progression of NAFL/NASH, but there are still several gaps that need to be filled in terms of their mode of action and specific mechanisms.

A variety of stresses can lead to the accumulation of unfolded and/or misfolded proteins in the endoplasmic reticulum (ER), resulting in ER stress and the subsequently unfolded protein reaction (UPR). The intestinal epithelium, a key barrier and messenger between the intestinal environment and the host immune system, which is susceptible to ER stress (Adolph et al., 2012). Chen et al. (2022) found that the induction of ER stress in intestinal mucosal epithelial cells could significantly disrupt the intestinal mucosal barrier and result in enteric dysbacteriosis, whereas the inhibition of ER stress with Pterostilbene significantly reversed these effects. A Winnie mouse model carrying a single missense mutation in the Muc2 mucin gene, characterized by reduced goblet cells and mucus layer and increased epithelial permeability, provided important evidence to elucidate that protein misfolding and the associated UPR pathway in secretory cells are major trigger for colitis (Das et al., 2013). Importantly, Winnie mice responded to anti-inflammatory agents, namely, glucocorticoids (Das et al., 2013), and azathioprine (Oancea et al., 2017), accompanied by a reduction in ER stress and UPR activation, a restoration of mature mucin production, and an amelioration of goblet cells failure. Notably, ER stress in the intestinal epithelium induces a series of adverse cellular responses, including redox imbalance, impaired autophagic fluxes, uncontrolled inflammatory responses, and apoptosis, which are involved in intestinal barrier dysfunction pathogenesis (Holczer et al., 2015; Vancamelbeke and Vermeire, 2017). However, some researchers have also found that the alleviation of protein misfolding and restoration of ER homeostasis can also lead to intestinal inflammation (Yoshida et al., 2001). Although both secretory cell ER stress and intestinal flora dysbiosis can trigger inflammation, the relative roles of the two in driving colitis progression remain unclear. Therefore, therapeutic strategies targeting ER stress may hold promise for mitigating intestinal barrier damage and intestinal disease. Nanoparticles can be synthesized in short steps and often use polyethylene glycol (PEG) and polycaprolactone (PCL) FDA approved polymers with high drug loading capacities to resolve these challenges, and effective in specific drug delivery, diagnosis and therapy in clinic (Verma et al., 2021).

IRE1α-XBP1 is the most evolutionarily conserved UPR pathway, and is closely associated with insulin resistance, dyslipidemia, hepatic steatosis and NAFLD inflammation (Lebeaupin et al., 2018). Following ER stress, the ribonucleic acid endonuclease activity of IRE1α cleaves X-box binding protein 1 (XBP1) mRNA to produce splice osomal XBP1 mRNAs (XBP1s). XBP1s is a key transcription factor of ER stress and is involved in the constitutive maintenance of core genes of ER in almost all cells as well as in the transcriptional regulation of a range of condition-specific target genes (Shaffer et al., 2004; Acosta-Alvear et al., 2007). It is also required for the development and survival of the secretory cell. Kaser et al. (2008) found that XBP1 deficiency in intestinal epithelial cells (IECs) resulted in spontaneous enterocolitis and increased the susceptibility of IECs to colitis secondary to Paneth cell defects and IBD inducers. Interestingly, single-nucleotide polymorphisms (SNPs) in the XBP1 gene significantly increase the risk of Crohn's disease and ulcerative colitis (Kaser et al., 2008). Most importantly, Chen et al. (2021) reported that HFD-fed mice exhibited a reduced abundance of tight junction proteins (claudin-1, claudin-4, ZO-1, and occludin), while ER stress-associated proteins, such as p-IRE1α and BIP, was activated, and Gly supplementation improved the intestinal mucosal barrier in HFD-induced obese mice by reducing ER stress-related signaling. Therefore, the present study was proposed to explore whether XBP1s plays an important role in preventing the progression of NASH via modulating dysbacteriosis and intestinal barrier damage by constructing *Xbp1* siRNA loading TPGS nano-micelles (hereafter named FT@XBP1).

## Materials and methods

### Fabrication of FT@XBP1

Rhodamine B (RhB) isothiocyanate DMSO solution (0.266 mg/ml, 500 μl) was added into the enzyme-free aqueous solutions [1.65 mg/ml, 1 ml, *n*(RhB): *n*(si*Xbp1*) = 2:1] and shaken well. NaHCO_3_ solution (0.1 ml, 1 M) was added to adjust pH to 8.0 and then stirred overnight. The mixture was dialyzed using deionized H_2_O (9 L, three times) and lyophilized to obtain rhodamine B modified si*Xbp1* (RhB-si*Xbp1*), and then stored at −20°C. Folic acid modified TPGS delivery system was prepared as follow. Briefly, the functional FA-TPGS@RhB-si*Xbp1* (herein called FT@XBP1) was synthesized by the cross-linking effect [*n*(FA-TPGS): *n*(si*Xbp1*) = 5:1]. Firstly, FA-TPGS (2.24 mg), RhB-si*Xbp1* and 1,4 dioxane (2 ml) were added into a penicillin bottle. The samples were sonicated for 20 min and then assembled using a micro flow meter (7 ml ultrapure water, 2 h with rapid stirring). And the nanocarriers were dialyzed after assembly. Then products were lyophilized for 24 h and stored at −20°C for further characterizations.

### Mice and treatment

Male C57BL/6J mice at the age of 5–7 weeks (18–20 g) were purchased from Gem Pharmatech corporation (Jiangsu, China). Mice were housed in specific pathogen-free (SPF) conditions and fed with either a chow diet (CD) or a fat-, fructose- and cholesterol-rich (FFC) diet for 12 and 24 weeks, respectively, to obtain the different stages of NAFLD, and NASH mouse models were established by feeding an FFC diet for 24 weeks. Mice in the NASH group were randomly divided into two subgroups (FFC and FFC + FT@XBP1) at the beginning of 20 weeks. FT@XBP1 were injected intravenously (2 OD, 0.05 nM, 100 μl) every 3 days for 4 weeks through the tail vein.

The body weights were recorded once a week during the experiments. Fecal samples were collected from each mouse at week of 0, 12 and 24, and quickly placed into liquid nitrogen for 15 min and then stored at −80°C for 16S rDNA gene sequencing. Mice were euthanized after 12 and 24 weeks of FFC diet feeding, and blood samples were collected by removing the eyeballs, allowing them to clot for at least 30 min at room temperature, and then centrifuged at 3,000 rpm for 10 min to collect the serum, which was stored at −80°C. The liver was isolated and weighed; then, a portion of the liver tissue was fixed in a 4% paraformaldehyde solution and embedded in paraffin. Another portion of the liver tissue was quickly frozen in liquid nitrogen and embedded in an optimal cutting temperature compound (OCT) for frozen sectioning; the remaining liver tissue was cut into small pieces and quickly placed into liquid nitrogen for further RNA and protein extraction. The epididymal fat tissue was isolated, weighed, and stored at −80°C. The colon tissue was isolated and washed with physiological saline to remove intestinal contents before being quickly placed in liquid nitrogen and stored at −80°C in a freezer. All animal experiments were performed according to procedures approved by the Laboratory Animal Ethics Committee of Anhui Medical University (LLSC20221000).

### Liver and intestinal tissue histopathological analysis

Mouse hepatic and intestinal samples were fixed in 4% paraformaldehyde for 12 h and embedded in paraffin. Paraffin-embedded samples were sectioned and then subjected to hematoxylin and eosin (H&E) staining for the assessment of liver and intestine histopathology. Liver tissue embedded in OCT was sectioned into 8–10 μm slices, and Oil Red O staining was performed on these frozen sections to measure the hepatic steatosis according to manufacturer's protocol. Masson's trichrome staining and Sirius red staining were performed on 4-μm paraffin-embedded liver tissue sections to evaluate the degree of liver fibrosis according to the manufacturer's protocol. The NAFLD activity score (NAS) was calculated from the grade of steatosis, inflammation, and ballooning as previously reported (Kleiner et al., 2005). In brief, steatosis was quantified as 0 (<5%), 1 (5%−33%), 2 (>33%−66%), and 3 (>66%) based on the percentage of liver cells containing fat droplets. Lobular inflammation was scored as 0 (no foci), 1 (<2 foci), 2 (2–4 foci), and 3 (>4 foci) according to the inflammation foci per 200 × field. Ballooning degeneration was scored as 0 (none), 1 (few), and 2 (many) according to the number of ballooning hepatocytes. The hepatic and intestinal histology and NAS were independently evaluated by two pathologists who were blinded to the sample groups.

### Immunofluorescence assay

Fresh liver tissues were embedded with OCT and cut into 8–10 μm slices. Then, the sections were washed with PBS and blocked with 2% BSA at room temperature for 1 h. The sections were then co-incubated with various primary antibodies at 4°C overnight in a light-avoiding environment. Subsequently, the sections were washed using PBS twice and co-incubated with a secondary antibody at 37°C in dark for 1 h. Finally, the sections were washed with PBS and stained with DAPI for 5 min, and photographed using a fluorescence microscope.

### Detection of serum lipopolysaccharide and serum parameters

Serum alanine aminotransferase (ALT), aspartic transaminase (AST) and total cholesterol (TC) in mice were detected using an Mindray BS-430 automated biochemical analyzer. The levels of hepatic triglycerides (TG) and lipopolysaccharides (LPSs) in mouse serum were detected according to the manufacturer's instructions (ADS Bio, Jiangsu, China).

### 16s rDNA gene sequencing and bioinformatics analysis

The feces of seven mice were collected for 16S rDNA sequencing analysis. In brief, total bacterial genomic DNAs were extracted using MagPure Soil DNA LQ Kit (D6356-02, Magen) following the manufacturer's instructions. The quality of the DNA was verified using agarose gel and quantified using a NanoDrop 2000c spectrophotometer (Thermo Fisher Scientific, United States), and then stored at −20°C until further processing. The diluted DNA was used as template for the PCR amplification of bacterial 16S rDNA genes with the bar-coded primers and Takara Ex Taq (Takara). The primers were 343F (5′-TACGGRAGGCAGCAG-3′) and 798R (5′-AGGGTATCTAATCCT-3′). Amplicon quality was visualized using gel electrophoresis, purified with AMPure XP beads (Agencourt), and amplified for another round of PCR. After being purified with the AMPure XP beads again, the final amplicon was quantified using a Qubit dsDNA assay kit. Equal amounts of purified amplicon were pooled for subsequent sequencing.

The 16S amplicon sequencing and analysis were conducted by OE biotech Co., Ltd. (Shanghai, China). In brief, raw sequencing data were in FASTQ format. Paired-end reads were then preprocessed using Cut adapt software to detect and cut off the adapter. After trimming, paired-end reads were filtered for low-quality sequences, denoised, merged and detect, and the chimera reads were cut off using DADA2 with the default parameters of QIIME2 (2020.11). Finally, the software outputs the representative reads and the ASV abundance table. The representative read of each ASV was selected using QIIME2 package. All representative reads were annotated and blasted against Silva database Version 138 (or Unite; 16S rDNA) using q2-feature-classifier with the default parameters.

### RNA extraction and qRT-PCR

Approximately 1 ml of TRIzol (R0016, Beyotime Biotechnology, China) was added to 20 mg of liver tissues and the total RNA was extracted according to the manufacturer's instructions. The total RNA (1 μg) was reverse-transcribed into cDNA using PrimeScript™RT Master Mix (RR036Q, Takara Bio, Japan). Real-time quantitative polymerase chain reaction (qRT-PCR) was performed using the QuantiNova SYBR Green PCR Kit (208054, QIAGEN, Germany) and the Light Cycler^®^ 96 Real-time PCR System (Roche, Switzerland), with the β-actin gene as the internal control. The relative expression of each gene mRNA was calculated using the 2^−Δ*ΔCt*^ method. Detailed information about the sequence used in this study is listed in Supplementary Table 1.

### Western blot analysis

Approximately 5 mg of liver or colon tissues were weighed and added to 1 ml of protein lysis buffer containing 1% PMSF (protease inhibitor). The mixture was homogenized using a mini-bead beater for 30 s, then lysed on ice for 30 min, and centrifugated at 12,000 rpm for 10 min to collect the protein supernatant. The total protein concentration was quantified using the BCA assay kit before mixing with 5 × loading buffer and boiling for 10 min in boiling water. Next, 20–30 μg of each sample was added to the polyacrylamide gel and subjected to electrophoresis, then, the protein was transferred onto PVDF membrane, and blocked with 5% milk at room temperature for 2 h. After washing, the membrane was incubated overnight with corresponding primary antibodies (such as β-actin, XBP1s, Col1αI, and α-SMA, all diluted at 1:1,000). Finally, the membrane was incubated with secondary antibodies and visualized using an Image Quant LAS 4000 chemiluminescence imaging system. Detailed information about the antibodies used in this study is shown in Supplementary Table 2.

### Statistical analysis

Statistical analysis was performed using SPSS 16.0, and all data were presented as mean ± standard deviation (SD). The differences between two groups were analyzed using Two-tailed Student's *t*-test, while one-way ANOVA was used to analyze the differences between more than two groups. Bonferroni's *post hoc* test was used for multiple comparisons. A *p-*value of <0.05 was considered to be statistically significant.

## Results

### FFC diet induces NASH and liver injury

**Figure 1:**
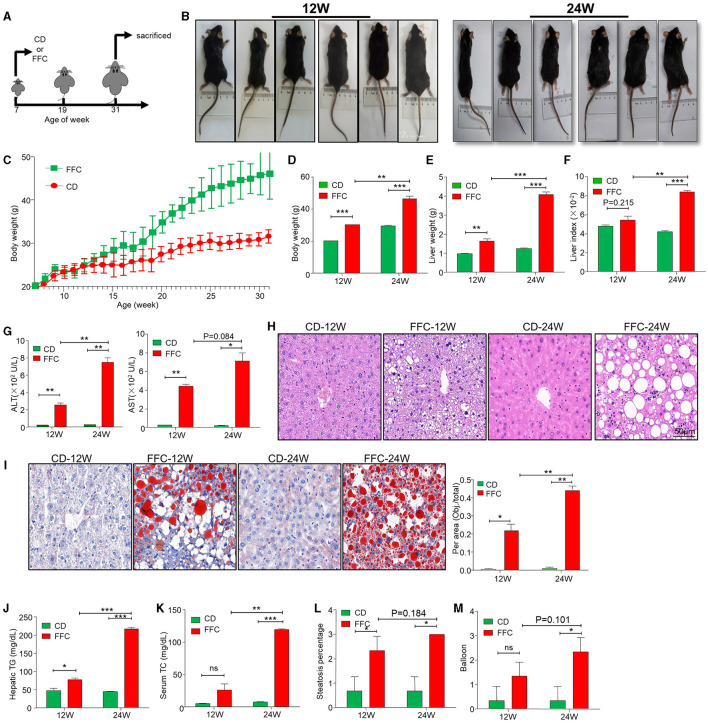
FFC diet exacerbated hepatic steatosis and NASH formation in mice. **(A)** Schematic overview of experimental design. **(B)** Images of mice fed with chow- (CD) or FFC- diet for 12 or 24 weeks. Body weights (C, D), liver weights **(E)**, liver/body weight ratio **(F)**, and serum ALT and AST **(G)** in mice fed on CD or FFC diet for 12 and 24 weeks. **(H)** H&E, **(I)** Oil red O staining and **(J)** hepatic TG of hepatic tissues from CD or FFC diet-fed mice for 12 and 24 weeks (scale bar = 50 μm). FFC diet increased the levels of serum TC **(K)**, and aggravated hepatic steatosis **(L)** and balloon **(M)**. Data are presented as the means ± SD (error bar) with *p < 0.05, **p < 0.01, and ***p < 0.001 vs. indicated group (*n* = 3 for each group). FFC, high-fat high-cholesterol; ALT, alanine transaminase; AST, aspartate transaminase; CD, chow diet.

To mimic the different stages of NAFLD in humans, the two groups of mice were fed a high-fat, high-sugar, and high-cholesterol (FFC) diet for 12 and 24 weeks, while control mice were given a chow diet (CD) feeding (Figure 1A). Body weights were measured once a week, as shown in Figures 1B–D. After 12 weeks of FFC dietary (hereafter named FFC_12W) intervention, the body weight increased significantly compared with that in the CD group, and FFC dieting for 24 weeks (hereafter named FFC_24W) increased the body weight even more (*p* < 0.05). Consistently, we found that the liver weight and the liver index (liver-to-weight ratio) were significantly higher than those in the CD diet group (Figures 1E, F, *p* < 0.01). The levels of serum alanine aminotransferase (ALT) and aspartate aminotransferase (AST) were also significantly elevated in mice fed with FFC diet (Figure 1G). HE staining showed that FFC_24W significantly increased fatty deposits in the liver, as demonstrated by macrovesicular and microvesicular steatosis, massive infiltration of inflammatory cells in the central venous area and the confluent area, and significant ballooning and fat accumulation in some hepatocytes compared with mice fed with CD or FFC_12W (Figure 1H). Oil red O staining further confirmed that hepatic steatosis was significantly increased in FFC-fed mice compared to the CD group and was more pronounced in the FFC_24W group (Figure 1I). In addition, we found that the contents of hepatic TG (Figure 1J) in the FFC_24W group were significantly higher than in the FFC_12W group (217.20 ± 7.94 nmol/mg vs. 77.30 ± 8.13 nmol/mg, *p* < 0.001). Similarly, the levels of serum triglyceride (Figure 1K), hepatic steatosis (Figure 1L) and significant ballooning (Figure 1M) in some hepatocytes compared with mice fed with CD or FFC_12W were also significantly increased in the FFC_24W group. Taken together, our results suggested that mice fed with FFC dieting for 24 weeks caused significant fat deposition and injury in the liver.

### FFC diet promotes liver fibrosis in NASH model

**Figure 2:**
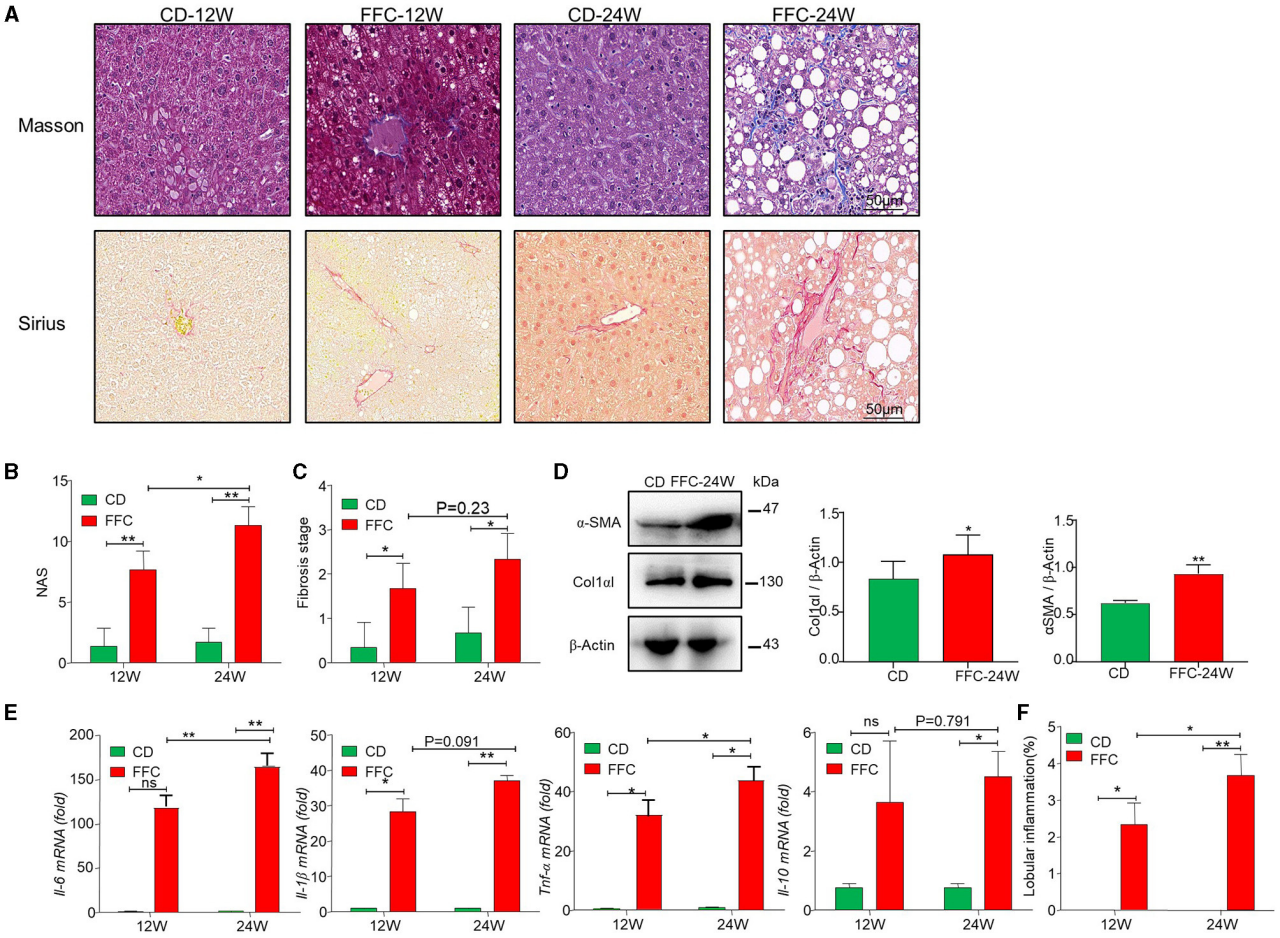
FFC diet promoted fibrosis in NASH mice model. **(A)** Representative Masson (upper panel) and Sirius red (lower panel) staining images (scale bar = 50 μm), and NAS **(B)** and fibrosis stages **(C)** of liver tissues from mice in the CD and FFC group (*n* = 3). **(D)** The expression of fibrotic proteins of α-SMA and Col1αI in liver tissues, and semi-quantitatively analyzed the band intensity (*n* = 3). **(E)** Inflammatory factors *Il-6, Il-1*β*, Tnf-*α, and *Il-10* in hepatic tissues of FFC fed mice or relative controls were measured using qRT-PCR, and **(F)** the lobular inflammation was calculated (*n* = 3 for each group). Data are presented as the means ± SD (error bar) with *p < 0.05 and **p < 0.01 vs. indicated group (*n* = 3 for each group). NAS, NAFLD activity score.

Fibrosis is a serious consequence secondary to chronic liver injury and inflammation and a risk factor for NASH worsening. In the current study, we used Sirius and Masson staining to determine hepatic collagen deposition and found that collagen deposition and pericellular fibrosis were significantly increased in the livers of FFC-fed mice compared to CD-fed mice (Figure 2A). The NAS was used for the quantitative evaluation of unique NASH-identified lesions. We found that both the FFC_24W and FFC_12W groups significantly increased NAS as demonstrated by hepatic steatosis, pronounced inflammation and balloon-like degeneration compared to mice in the CD group, and mice in the FFC_24W group showed higher NAS than those in the FFC_12W group (Figure 2B). The fibrosis index was calculated based on Masson and Sirius staining, and as shown in Figure 2C, FFC dietary feeding significantly elevated the fibrosis index compared to CD group, but there was no significant difference between the FFC_24W and FFC_12W groups (*p* = 0.23). Immuno-blotting assays also revealed that the expression levels of both α-SMA and Col1αI protein (two fibrosis-related markers) in liver tissues were significantly higher in FFC_24W group compared with the normal group (Figure 2D).

Inflammatory cell infiltration is another major pathological feature of NAFLD (Sacks et al., 2018). Therefore, we examined the mRNA levels of several inflammatory factors using liver tissues via qRT-PCR assay, and we found that FFC feeding significantly increased the expression of pro-inflammatory factors *Il-6, Il-1*β, and *Tnf-*α and anti-inflammation factor *Il-10* compared to mice fed with CD diet (Figure 2E). Moreover, the levels of pro-inflammatory factors (*Il-6, Il-1*β, and *Tnf-*α) were significantly elevated in FFC_24W group compared with the FFC_12W group, but there was no significant difference between the FFC_24W and FFC_12W groups in *Il-10* levels. Similarly, FFC_24W significantly increased lobular inflammation compared to mice in FFC_12W and CD groups (Figure 2F). These results suggested that FFC dietary feeding increased hepatic inflammation and promoted the progression of NASH.

### Dynamic changes of intestinal flora in the development of NASH

To assess the dynamic changes of the gut microbiota at different stages of NAFLD, next-generation sequencing was used to sequence the V3–V4 region of the gut bacterial 16S rDNA gene in the feces of mice from the CD, FFC_12W and FFC_24W groups (*n* = 7). A total of 1,680,254 raw reads were obtained, and 131,178 high-quality reads were filtered, from which a total of 5,070 ASVs were clustered based on 99% sequence similarity. Observed-species rarefaction curves (Supplementary Figure 1A) and Shannon (Supplementary Figure 1B) showed that the current sequencing depth was sufficient to capture the majority of gut microbiota in all samples.

As shown in Supplementary Figure 2A, the ACE index, and Chao1 index were higher in the FFC diet group compared to the CD group, and the Shannon index and Chao1 index were higher in the FFC_24W group compared to the FFC_12W group, but there was no statistical significance among the three groups. According to the relative abundance of ASVs, principal component analysis (PCA) and Bray-Curtis distance-based principal coordinate analysis (PCoA) were used to analyze the structural changes in the gut microbiota in each sample, and the results showed that the gut microbiota of FFC-fed mice were completely separated from that of CD-fed mice (Supplementary Figures 2B, C). In addition, PCoA (Supplementary Figure 2C) similarity analyses showed that there was a complete separation of the gut flora in the FFC_12W and FFC_24W group (*p* = 0.001).

**Figure 3:**
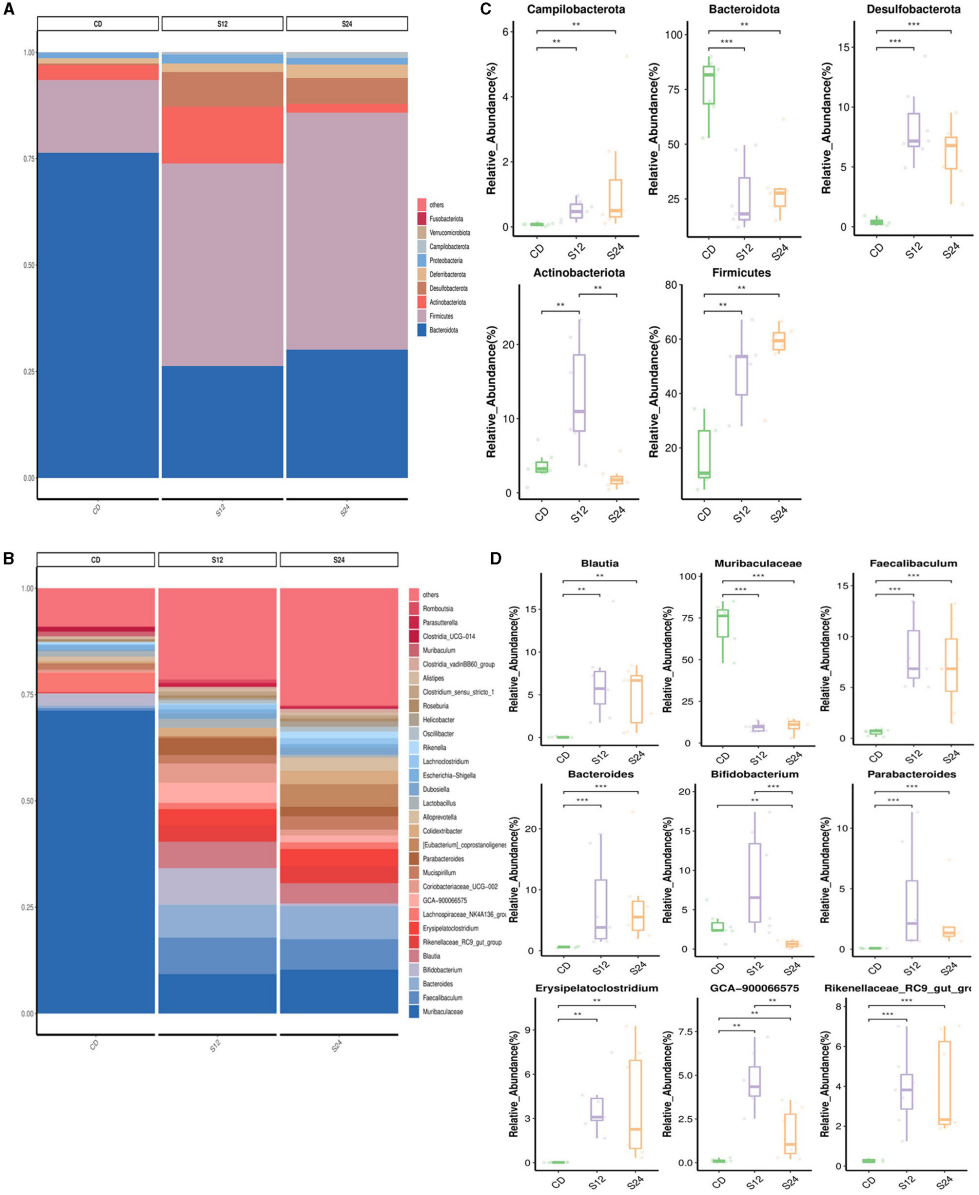
The alteration in the gut microbiome associated with FFC diet. Relative abundance of bacterial communities from mice at the different stages of NASH at the phylum **(A)** and genus level **(B)**. Comparison of average abundance of specific gut bacteria from CD diet or FFC-diet for 12 or 24 weeks at the phylum **(C)** and genus level **(D)**. Data are presented as the means ± SD (error bar) with **p < 0.01, and ***p < 0.001 vs. indicated group (*n* = 7).

The relative abundance of gut microbiota in each group at the phylum and genus levels is shown in Figures 3A, B. At the phylum level, the gut microbiota composition in the FFC_12W group, compared to the CD group, significantly changed, as shown by a higher abundance of Campilobacterota (0.07% vs. 1.09%, *p* < 0.001), Desulfobacterota (4.1% vs. 8.4%, *p* < 0.001), Actinobacteria (3.6% vs. 13.1%, *p* < 0.01) and Firmicutes (17.2% vs. 47.9%, *p* < 0.01), whereas the abundance of Bacteroidota (76.1% vs. 25.7%, *p* < 0.001) significantly decreased (Figure 3C). The relative abundance of Firmicutes (25.7% vs. 56.1%, *p* > 0.05) was further upregulated in the FFC_24W group compared to the FFC_12W group, whereas the relative abundance of Actinobacteriota (13.1% vs. 2.1%, *p* < 0.001) was significantly lowered (Figure 3C).

At the genus level, the relative abundance of Blautia (0.05% vs. 6.68%, *p* < 0.001), Faecalibaculum (0.61% vs. 8.33%, *p* < 0.001), Bacteroides (0.60% vs. 7.37%, *p* < 0.001), Bifidobacterium (2.94% vs. 8.53%, *p* < 0.05), Parabacteroides (0.07% vs. 3.83%, *p* < 0.001), Erysipelatoclostridium (0.02% vs. 3.81%, *p* < 0.01), GCA-900066575 (0.10% vs. 4.66%, *p* < 0.01), and Rikenellaceae_RC9_gut_group (0.26% vs. 3.86%, *p* < 0.001) in the FFC_12W group was significantly increased compared to CD group; however, the relative abundance of Muribaculaceae was significantly reduced (70.89% vs. 9.34%, *p* < 0.001; Figure 3D). In comparison with the FFC_12W group, the abundance of Blautia (6.68% vs. 6.88%, *p* > 0.05) and Bacteroides (7.37% vs. 7.61%, *p* > 0.05) was further increased, while the relative abundance of two intestinal probiotics, Bifidobacterium (8.53% vs. 0.63%, *p* < 0.001) and GCA-900066575 (4.66% vs. 1.63%, *p* < 0.01) was significantly decreased (Figure 3D).

### FT@XBP1 ameliorated hepatic injury and fibrosis in the NASH model

**Figure 4:**
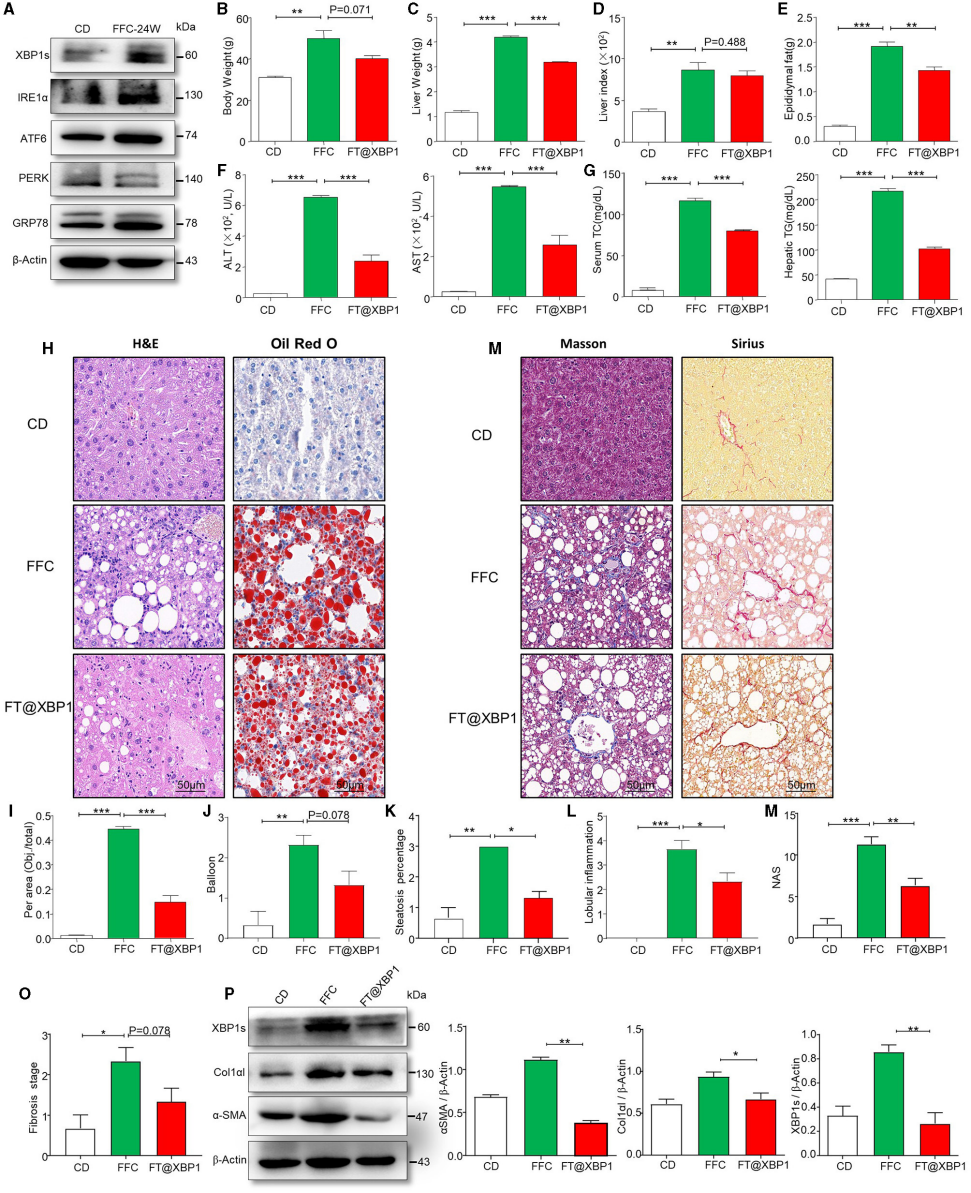
FT@XBP1 alleviated liver injury, steatosis and fibrosis in NASH mice. **(A)** Western blot analysis determined the expression of XBP1s, IRE1α, ATF6, PERK, and GRP78 in liver tissues from FFC diet induced NASH mice. **(B)** Body weights, **(C)** liver weights, **(D)** liver/body weight ratio, **(E)** epididymal fat, **(F)** serum ALT and AST, **(G)** and serum TC and hepatic TG in FFC-fed or chow-fed mice, and FFC-fed mice treated with FT@XBP1. **(H)** Representative images of H&E (left panel), Oil red O staining (right panel) in hepatic tissues from mice fed with FFC diet and FFC-fed mice treated with FT@XBP1, and **(I)** semi-quantitative analyzed the staining intensity of Oil red O staining (scale bar = 50 μm). FT@XBP1 treatment alleviated FFC diet induced **(J)** balloon, **(K)** steatosis percentage and **(L)** lobular inflammation. **(M)** Representative images of Masson (left panel), Sirius (right panel) staining in hepatic tissues from mice fed with the FFC diet and FFC-fed mice treated with FT@XBP1 (scale bar = 50 μm). FT@XBP1 treatment decreased **(N)** NAS and **(O)** fibrosis stages, and **(P)** decreased the expression of XBP1s, α-SMA and Col1αI in liver tissues and semi-quantitative analysis of figure **(P)**. Data are presented as the means ± SD (error bar) with *p < 0.05, **p < 0.01, and ***p < 0.001 vs. indicated group (*n* = 3 for each group).

ER stress plays an important role in the occurrence and development of NAFLD (Zheng et al., 2022). We used an immunoblotting assay to detect the expression of ER stress-related proteins, and found that GRP78, ATF6, PERK, IRE1a, and XBP1s in the liver of FFC diet-fed mice were significantly upregulated compared to CD mice (Figure 4A, Supplementary Figure 3A). Moreover, ER stress downstream transcription factor XBP1s was more significantly upregulated than other ER stress markers (Figure 4A). In addition, we used immunofluorescence (IF) assays to confirm that XBP1s expression was higher in the liver tissue of FFC diet-induced NASH mice than in CD-fed mice (Supplementary Figure 3B), suggesting that XBP1s may play an important role in NASH progression. Thus, we used folate to modify TPGS and incorporate *Xbp1* siRNA (FT@XBP1) to knock down XBP1 and found that the body weight (Figure 4B), and liver weight (Figure 4C) were significantly reduced in mice treated with FT@XBP1 than in mice fed with FFC diet, but had no effect on the liver-to-weight ratio compared with FFC diet-fed mice (Figure 4D). We also found that FT@XBP1 could significantly reduce epididymal fat in mice fed with FFC diet (Figure 4E). Moreover, FFC diet-induced mice had high levels of serum alanine transaminase (ALT) and aspartate aminotransferase (AST) in comparison with mice with the CD diet, and FT@XBP1 treatment remarkably decreased ALT and AST levels (Figure 4F). Most interestingly, we also found that FT@XBP1 treatment could significantly reduce FFC diet-induced serum TC and hepatic TG compared to mice fed with FFC diet (Figure 4G).

We also investigated the role of XBP1s on liver injury, lipid accumulation, inflammatory cells infiltration, collagen deposition, and fibrosis using HE staining, Oil red staining, Masson staining, and Sirius staining, respectively. We found that XBP1 deficiency largely attenuated FFC diet-induced liver injury (Figure 4H) and steatohepatitis-related parameters in mice, for example intrahepatic ballooning (Figures 4H–J) and steatosis percentage (Figure 4K), which relieved lipid accumulation and improved liver histology. As previously reported, inflammatory cell infiltration plays a vital role in NASH progression. Interestingly, a decrease in lobular inflammation was also noted in FT@XBP1*-*treated mice compared to FFC-fed mice (Figure 4L).

As fibrosis is a vital risk for the deterioration of NASH to hepatic fibrosis, we determined collagen deposition in the liver tissues using Masson and picrosirius red staining (Figure 4M). Most interestingly, we found that the livers of mice fed with FFC diet demonstrated an evident increase in collagen deposition and pericellular fibrosis compared with CD-fed mice, and these pathological changes were significantly reversed by treating mice with FT@XBP1 (Figure 4M). The NAS score was used for the quantitative evaluation of unique NASH-identified lesions. As shown in Figure 4N, the FFC diet significantly increased NAS compared with the CD diet, whereas FT@XBP1 treatment remarkably reduced NAS compared with mice fed with an FFC diet, as demonstrated by evidently reduced lobular inflammation and steatosis. Consistently, we found that FT@XBP1 treatment remarkably reduced the fibrosis stage (Figure 4O). In addition, indicators of hepatic fibrosis, such as Col1αI and α-SMA, were significantly reduced in mice treated with FT@XBP1 compared with FFC diet-fed mice, as demonstrated by Western blot analysis (Figure 4P). These results suggested that FT@XBP1 treatment ameliorated steatohepatitis and fibrosis in response to FFC diet-induced NASH.

### FT@XBP1 partially restored the dysbiosis of the gut microbiota in NASH mice

To explore the effect of FT@XBP1 treatment on the diversity of gut microbiota in NASH mice, the V3–V4 regions of the intestinal bacterial 16S rDNA genes from CD, FFC_24W, and FFC diet in combination with FT@XBP1 (120 mg/kg) treatment were sequenced using the next-generation sequencing. A total of 1,681,217 raw reads were obtained, and 1,303,526 high-quality reads were filtered, of which a total of 4,997 differential ASVs were clustered based on 99% sequence similarity. Observed-species rarefaction curves (Supplementary Figure 4A) and Shannon (Supplementary Figure 4B) reached a plateau, indicating that the current sequencing depth was reasonable and sufficient to capture most of the gut microbes in the samples. As shown in Supplementary Figure 5A, the Shannon index and Simpson index were slightly elevated in the FFC_24W and FT@XBP1 treatment groups, while the ACE index and Chao1 index were slightly reduced compared to the chow diet group but did not reach a statistically significant difference. Beta diversity represents the difference in the composition of microbial communities between samples. In the current study, PCA (Supplementary Figure 5B) and PCoA (Supplementary Figure 5C) similarity analyses were used to observe the similarity of the species composition between samples. It was found that the gut microbiota of FFC-fed mice was completely separated from that of CD-fed mice (*p* = 0.001), but the gut microbiota of the FFC-fed mice was not completely separated from the FT@XBP1 treatment group, suggesting that FT@XBP1 treatment could partially restore the composition of the intestinal microbiota in NASH mice.

**Figure 5:**
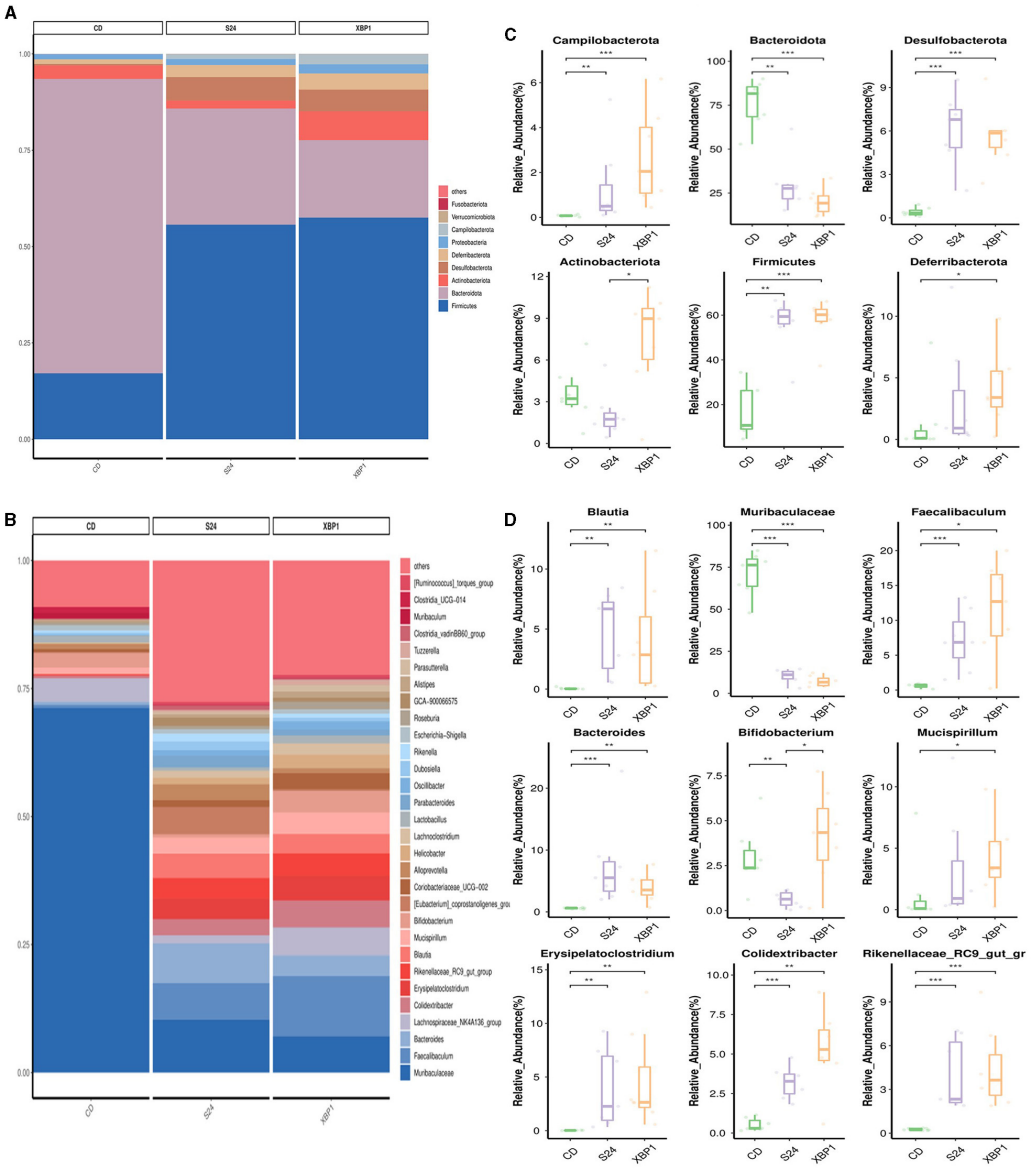
FT@XBP1 treatment partially restored the gut microbiome in FFC induced NASH model. Relative abundance of bacterial communities from mice at the phylum **(A)** and genus species level **(B)** in FFC diet fed mice or mice treated with FT@XBP1. Comparison of average abundance of specific gut bacteria from FFC diet fed mice or mice treated with FT@XBP1 at the phylum **(C)** and genus level **(D)**. Data are presented as the means ± SD (error bar) with *p < 0.05, **p < 0.01, and ***p < 0.001 vs. indicated group (*n* = 7).

The relative abundance of intestinal bacteria in each group at the phylum and genus levels is shown in Figures 5A, B. As shown in Figure 5C, at the phylum level, the relative abundance of Campilobacterota (0.07% vs. 1.33%, *p* < 0.001), Desulfobacterota (0.41% vs. 6.12%, *p* < 0.001) and Firmicutes (17.22% vs. 56.12%, *p* < 0.01) in the FFC_24W group was significantly increased compared with the CD group, while the relative abundance of Bacteroidota (76.06% vs. 29.53%, *p* < 0.01) and Actinobacteriota (3.57% vs. 2.09%, *p* > 0.05) was decreased significantly. FT@XBP1 treatment further increased the relative abundance of Campilobacterota (0.07% vs. 2.69%, *p* > 0.05) and Deferribacterota (1.35% vs. 3.21%, *p* < 0.05), but decreased the abundance of Desulfobacterota (6.12% vs. 5.66%, *p* > 0.05) compared to the FFC_24W group. Interestingly, we found that FT@XBP1 treatment significantly reversed FFC feeding induced decreases in Actinobacteriota abundance (2.09% vs. 7.42%, *p* < 0.05).

At the genus level (Figure 5D), FFC diet feeding significantly increased the relative abundance of Blautia (0.05% vs. 4.80%, *p* < 0.01), Faecalibaculum (0.61% vs. 7.20%, *p* < 0.001), Bacteroides (0.60% vs. 7.61%, *p* < 0.001), Erysipelatoclostridium (0.02% vs. 3.96%, *p* < 0.01), Colidextribacter (0.53% vs. 3.19%, *p* < 0.001) and Rikenellaceae_RC9_gut_group (0.26% vs. 3.99%, *p* < 0.001) compared to CD-fed mice, while the relative abundance of Muribaculaceae (70.89% vs. 10.22%, *p* < 0.001) and Bifidobacterium (2.94% vs. 0.63%, *p* < 0.01) was significantly decreased. FT@XBP1 treatment could further increase the abundance of Faecalibaculum (7.20% vs. 11.65%, *p* > 0.05), Rikenellaceae_RC9_gut_group (3.99% vs. 4.45%, *p* > 0.05) and Colidextribacter (3.19% vs. 5.29%, *p* > 0.05), while decreased the abundance of Muribaculaceae (10.22% vs. 7.09%, *p* > 0.05) compared to the FFC_24W group. More importantly, FT@XBP1 treatment not only effectively reversed the FFC_24W-induced decrease in intestinal probiotic Bifidobacterium (0.63% vs. 4.17%, *p* < 0.05), but also increased the intestinal abundance of Mucispirillum (3.21% vs. 4.25%, *p* > 0.05). These suggest that FT@XBP1 may prevent the progression of NASH by regulating the composition of gut microbiota.

In addition, we obtained functional annotations and relative abundance information of the gut microbiota in each group, and cluster analysis was performed according to the level of functional difference. As shown in Supplementary Figure 6, FFC diet feeding for 24 weeks showed significant activation of glycosaminoglycan degradation, glycosphingolipid biosynthesis-ganglio series, phenylpropanoid biosynthesis, sphingolipid metabolism and various types of N-glycan biosynthesis pathway, whereas FT@XBP1 treatment showed an opposite trend. Furthermore, we found that the apoptosis, ferroptosis and lysosome pathways were activated after 24 weeks of FFC dietary feeding, while FT@XBP1 treatment significantly inhibited the activation of these pathways. These results suggested that FT@XBP1 may play a vital role in the prevention of NASH progression by restoring the dysbiosis of the gut microbiota.

### FT@XBP1 ameliorates intestinal barrier dysfunction in NASH

**Figure 6:**
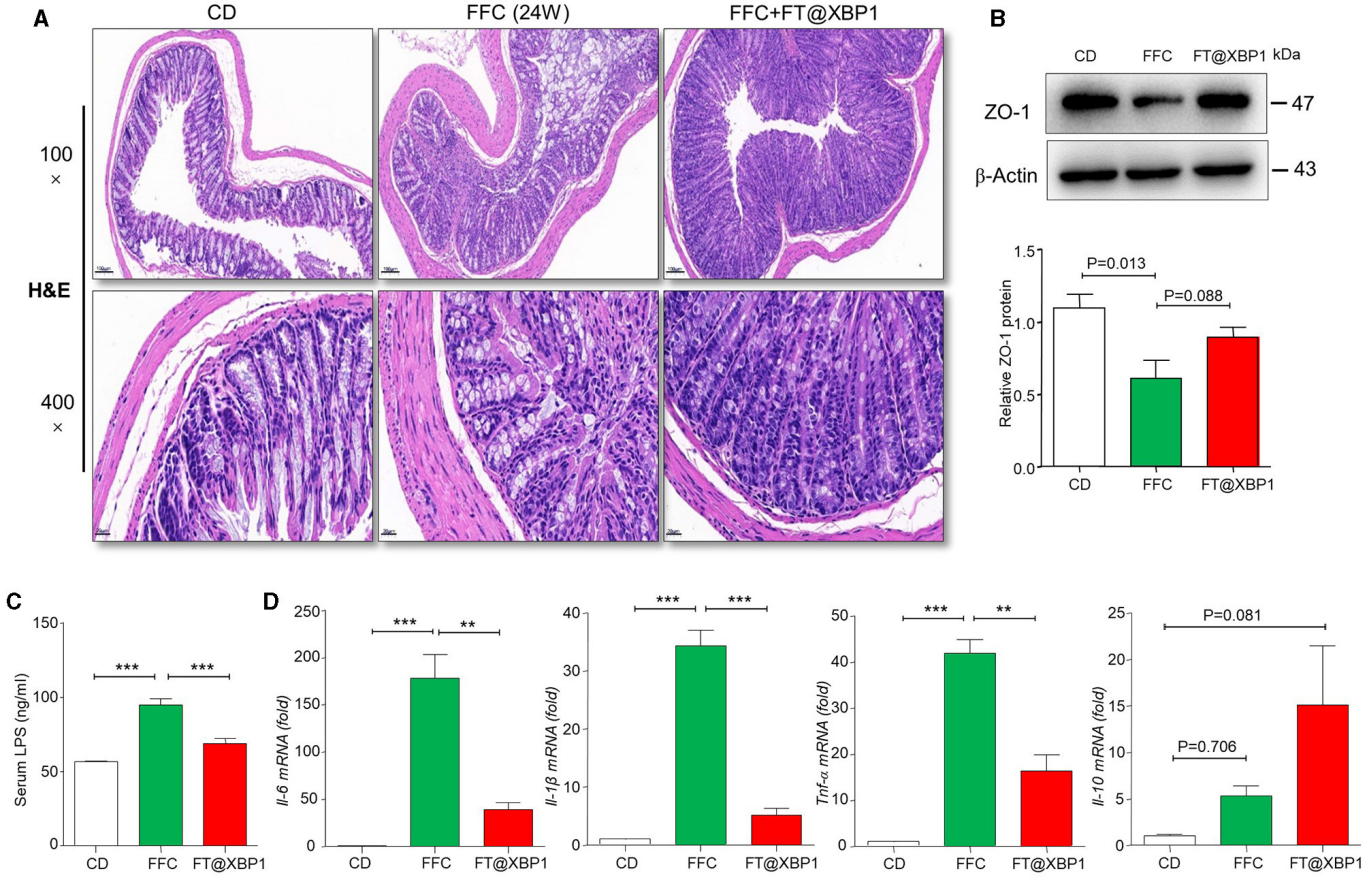
FT@XBP1 protected against intestinal barrier disruption and reduced serum LPS and inflammatory cytokines in NASH mice. **(A)** H&E staining of mouse intestinal tissue from CD, FFC and FT@XBP1 treatment group (scale bar = 50/20 μm). **(B)** Western blot analysis the expression of ZO-1 in colon tissues, and semi-quantitative analyzed. **(C)** FT@XBP1 treatment reduced the levels of serum LPS and **(D)** the inflammatory factors *Il-6, Il-1*β*, Tnf-*α, and *Il-10* in hepatic tissues in NASH mice. Data are presented as the means ± SD (error bar) with **p < 0.01, and ***p < 0.001 vs. indicated group (*n* = 3 for each group).

Intestinal mucosal homeostasis plays a vital role in maintaining the normal physiological activities of the organism, and intestinal mucosal barrier damage and increased intestinal epithelial permeability are often accompanied by the progression of NASH (Albillos et al., 2020). Thus, we first stained the mouse colonic mucosa using H&E staining, and found that FFC feeding significantly increased the degree of colonic epithelial barrier disruption (Figure 6A). Furthermore, the apical and crypt areas of colonic tissues showed significant structural disruption and disorganization, with intermediate deletions, while, the integrity of the colon barrier was found to be intact after 4-week intervention with FT@XBP1, which indicated that FT@XBP1 could ameliorate FFC diet-induced Intestinal mucosal barrier disruption (Figure 6A). Subsequently, we used immunoblotting assay to detect the expression level of epithelial tight junction protein 1 (zonula occludens-1, ZO-1), and found that the protein level of ZO-1 was significantly lower in FFC diet group compared with that of the CD diet group (Figure 6B). Furthermore, FT@XBP1 treatment induced a certain recovery of the ZO-1 protein compared with FFC group (Figure 6B), but there was no statistical difference (*p* = 0.088). To further understand the mechanism of FT@XBP1 on FFC-induced NAFLD progression, we examined the plasma endotoxin levels and found that plasma LPS levels were significantly elevated in the FFC dietary group compared with the CD group, whereas FT@XBP1 treatment obviously reversed this effect (Figure 6C). In addition, qRT-PCR assays showed that FT@XBP1 treatment significantly decreased the levels of inflammatory factors *Il-6, Il-1*β, and *Tnf-*α (Figure 6D), but the levels of *Il-10* were elevated to some extent compared with FFC diet mice (*p* = 0.081). Therefore, our results suggest that XBP1s plays an important role in maintaining intestinal mucosal homeostasis by regulating the expression of tight junction proteins, which together reduce the penetration of intestinal-sourced endotoxins into the circulation and then alleviate liver inflammation to prevent the progression of NASH.

## Discussion

The pathogenesis of NAFLD is complex, and the traditional “two-hit” hypothesis is not sufficient to fully explain the pathogenesis of NASH, especially for non-obese individuals (Wang et al., 2016), because there is evidence that TG is not hepatotoxic in mice with steatohepatitis (Yamaguchi et al., 2007). However, the accumulation of other lipids, such as free fatty acids, diacylglycerol, cholesterol, ceramides, and phospholipids, in the liver can induce ER stress, mitochondrial dysfunction, and oxidative stress, ultimately leading to liver inflammation and fibrosis (Feldstein et al., 2004; Tilg and Moschen, 2010). Recently, some researchers put forward a “multiple parallel hit model” (Tilg and Moschen, 2010; Papatheodoridi and Cholongitas, 2018), with intestinal flora disturbance and the related enterogenic endotoxemia possibly being the key “hit” leading to persistent liver injury and NAFLD progression (Poeta et al., 2017). NAFLD patients are often associated with varying degrees of intestinal flora disturbance and translocation, which manifests as decreased abundance of Bacteroidetes and an increased relative abundance of Firmicutes at the phylum level. Meanwhile, Gram-negative bacteria in the gut are overpopulated, while the abundance of Gram-positive bacteria such as Bifidobacterium and Lactobacillus are decreased significantly, and intestinal bacteria and LPS could translocate into the portal vein system, which results in enterogenic endotoxemia and persistent inflammatory injury in the liver (Zhu et al., 2013; Shen et al., 2017). Schnabl and Brenner (2014) found that mice lacking intestinal flora were resistant to the development of diet-induced liver steatosis; however, replenishing flora via fecal microbiota transplantation (FMT) can promote the development of NAFLD in these mice, and liver steatosis can be improved after application of probiotics and antibiotics. In the current study, we found that FFC diet significantly increased the relative abundance of Firmicutes and decreased the abundance of Bacteroidota compared to mice fed with chow diet at the phylum level. Meanwhile, we found that FFC diet increased the abundance of Blautia and Bacteroides, while the abundance of intestinal probiotics, such as Bifidobacterium and GCA-900066575, was significantly decreased at the genus level (Figure 3D). These results suggest that the development of NASH is accompanied by a disturbance of the intestinal flora.

Recently, the role of the intestinal mucosal barrier and inflammatory response in the development of NAFLD has been a high concern (Takaki et al., 2013; Tripathi et al., 2018). Dysbacteriosis and intestinal endotoxemia (IETM) are important causes of liver inflammation and NAFLD. The normal intestinal mucosal barrier can prevent the translocation and invasion of enterogenic pathogenic factors, such as intestinal bacteria and endotoxins (Brandl et al., 2017). However, in some pathological conditions, intestinal mucosal barrier integrality is impaired, and harmful substances such as bacterial DNA, lipopolysaccharide (LPS), and ethanol can enter the liver through the portal vein, thus promoting the progression of NASH (Pierantonelli et al., 2017; Longo et al., 2020). Germ-free animals transplanted with fecal from NAFLD patients demonstrated significant hepatic steatosis, multifocal necrosis, and inflammatory cell infiltration in the liver, accompanied by increased levels of serum LPS and inflammatory cytokines (such as IL-6 and MCP-1) and the disturbance of intestinal flora, indicating that the occurrence of NAFLD is closely related to intestinal flora disturbance (Chiu et al., 2017). In this study, we found that FFC diet not only impaired the integrity of intestinal mucosal barrier and decreased the levels of ZO-1 protein but also significantly increased the levels of several pro-inflammatory factors (such as IL6, IL-1β, and TNF-α) and plasma LPS compared to chow diet mice. These results suggest that high-fat diet may lead to enterogenic endotoxemia by altering the structure of the intestinal flora and aggravating liver inflammation, thus accelerating the progression of NASH.

The endoplasmic reticulum (ER) plays an essential role in protein folding and maturation, in addition to other metabolic processes. ER stress has been observed in nearly all chronic liver diseases and is distinguished by the activation of three UPR pathways. Guo et al. (2018) reported increased mRNA expression of ER stress markers, including BiP, activating transcription factor 4 (ATF4), and c/EBP-homologous protein (CHOP), in mice fed an HFD diet. Consistently, mice fed with the FFC diet demonstrated higher levels of ER stress-related proteins, including GRP78, PERK, ATF6, IRE1α and XBP1s, and IRE1α-XBP1s pathway was significantly elevated compared to other ER stress markers (Figure 4A). Dysregulation of the ER stress response has been implicated in intestinal inflammation associated with inflammatory bowel disease (IBD), a chronic condition characterized by changes in the mucosa and alteration of the gut microbiota (McGovern et al., 2010). However, the exact relationship between ER stress and gut microbes is largely unclear. In the current study, we found that FT@XBP1 treatment could not only significantly inhibit liver lipid accumulation and collagen deposition in the FFC diet fed mice but also improve high-fat-induced intestinal barrier dysfunction, as demonstrated by the restoration the integrity of the intestinal barrier (Figure 6A) and increased levels of ZO-1 protein expression (Figure 6B), which partially restored intestinal flora structure, reduced endotoxemia and alleviated liver inflammation. Similarly, Laudisi et al. (2019) demonstrated that mice fed a maltodextrin (MDX)-enriched diet exhibited an activated ER stress response in intestinal epithelial cells and an exacerbated intestinal inflammation, while treatment of mice with TUDCA prevented mucin-2 depletion and attenuated colitis in MDX-fed mice. Most interestingly, Grey et al. (2022) found that ERN2 was required for microbiota-induced goblet cell maturation and mucus barrier assembly in the colon, and mice lacking Ern2 had a dysbiotic microbial community. The secretory capacity of the gut epithelium, especially mucin and antimicrobial protein production, likely demands the maintenance of ER proteostasis (Kaser et al., 2008). These results suggested that ER stress transcription factor XBP1s evolved on mucosal surfaces to mediate crosstalk between gut microbes and the mucus barrier required for normal homeostasis and host defense. However, several pieces of evidence suggested that *Xbp1* deletion in mouse intestinal epithelial cells resulted in Paneth and goblet cell apoptosis, spontaneous ileal inflammation, and increased sensitivity to dextran sodium sulfate (DSS)-induced colitis (Kaser et al., 2008).

In summary, our study showed that the inhibition of XBP1s by FT@XBP1 could restore the integrity of the intestinal mucosal barrier and intestinal flora disturbance and inhibit LPS translocation, which subsequently reduces liver inflammation and lipid deposition and prevents NASH progression. However, this study inevitably has some limitations. First, this study established NASH model by feeding animals with FFC (high fat, high fructose and high cholesterol) diets. It was found that the progression of NASH induced by FFC diet was related to intestinal flora disturbance and the integrity of intestinal mucosal barrier, but the exact relationship was not fully understood. Second, intravenously administrated FT@XBP1 exerted its effect mainly by targeting the key transcription factor XBP1s, however, whether it affects the integrity of the intestinal mucosal barrier and regulates liver inflammation to indirectly restore intestinal microflora homeostasis in NASH is worthy of further study. Finally, the role of other downstream signaling pathways of ER stress in intestinal flora disturbance and the progression of NASH is also worth further investigation.

## Data availability statement

Data relating with the current paper are available upon reasonable request to the corresponding author. The 16S rDNA sequencing data was store in the SRA database (PRJNA1030503), with the following link https://www.ncbi.nlm.nih.gov/sra/.

## Ethics statement

The animal study was approved by the Laboratory Animal Ethics Committee of Anhui Medical University (LLSC20221000). The study was conducted in accordance with the local legislation and institutional requirements.

## Author contributions

MZ: Data curation, Writing – original draft, Formal analysis, Investigation, Methodology, Validation. YC: Investigation, Methodology, Writing – original draft, Project administration. YT: Methodology, Writing – original draft, Data curation, Formal analysis, Resources. SL: Data curation, Formal analysis, Methodology, Resources, Writing – review & editing. PR: Data curation, Formal analysis, Methodology, Writing – review & editing. GZ: Data curation, Formal analysis, Methodology, Writing – review & editing. LX: Writing – review & editing, Conceptualization, Funding acquisition, Resources, Supervision, Writing – original draft. JL: Conceptualization, Funding acquisition, Writing – original draft, Writing – review & editing, Data curation.
