# Supplementary material for: Nanoparticles alleviate non-alcoholic steatohepatitis via ER stress sensor-mediated intestinal barrier damage and gut dysbiosis
Source: Front Microbiol. 2024 Mar 7;14:1271835. doi: 10.3389/fmicb.2023.1271835 (PMC10956414; doi:10.3389/fmicb.2023.1271835)
Supplement: Supplementary file 1 [file Data_Sheet_1.pdf]

Supporting Materials

Folic acid modified TPGS nanoparticles alleviate non-alcoholic steatohepatitis via ER stress sensor XBP1s mediated intestinal barrier damage and gut dysbiosis

Contents of the Supporting Data

Contents	Page
Supporting Tables	Page 2
Supplementary Figure and legends	Page 3-6

Supporting Tables

Table 1 RNA sequence used in this paper

RNA	Sequence	
Mouse IL-6	Forwardprimer:	CAACGATGATGCACTTGCAGA
NM_001314054.1	Reverseprimer:	TGTGACTCCAGCTTATCTCTTGG
Mouse IL-1β	Forwardprimer:	TGCCACCTTTTGACAGTGATG
NM_008361.4	Reverseprimer:	TGATGTGCTGCTGCGAGATT
Mouse TNF-α	Forwardprimer:	ACCCTCACACTCACAAACCAC
NM_001278601.1	Reverseprimer:	ATAGCAAATCGGCTGACGGT
Mouse IL-10	Forwardprimer:	TGCAGTGTGTATTGAGTCTGCT
NM_010548.2	Reverseprimer:	GCTCTGTCTAGGTCCTGGAG

Table 2 Antibodies used in this study

Antibody	Source	Application
anti-mouse β-actin	Santa Cruz	WB
anti-rabbit XBP1s	Cell signal	WB
ant-rabbit XBP1®488	Santa Cruz	IF
anti-rabbit PERK	Cell signal	WB
anti-rabbit IRE1α	Cell signal	WB
anti-rabbit GRP78	Cell signal	WB
anti-rabbit ATF6	Cell signal	WB
anti-rabbit α-SMA	ZENBIO	WB
anti-mouse α-SMA	ZENBIO	IF, IHC
anti-rabbit F4/80	Cell signal	IF, IHC

Supplementary Figure and legends:

Supplementary Fig. 1

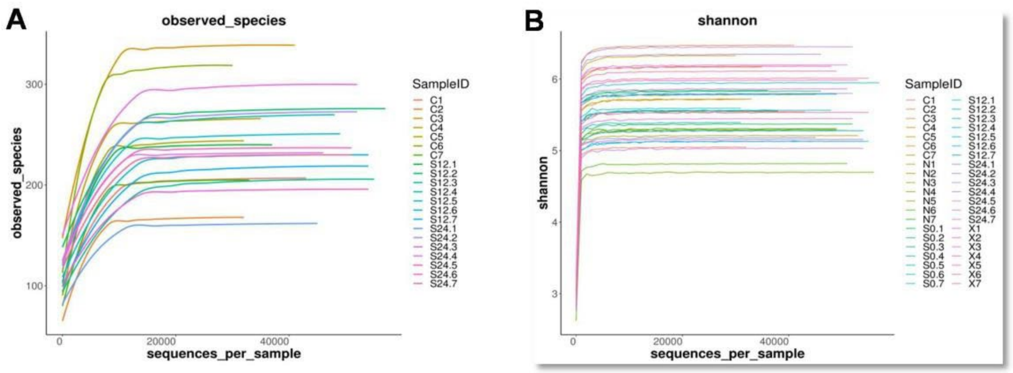

Figure S1. The rarefaction curve (A) and shannon curves (B) in mice at the different stages of NASH.

Supplementary Fig. 2

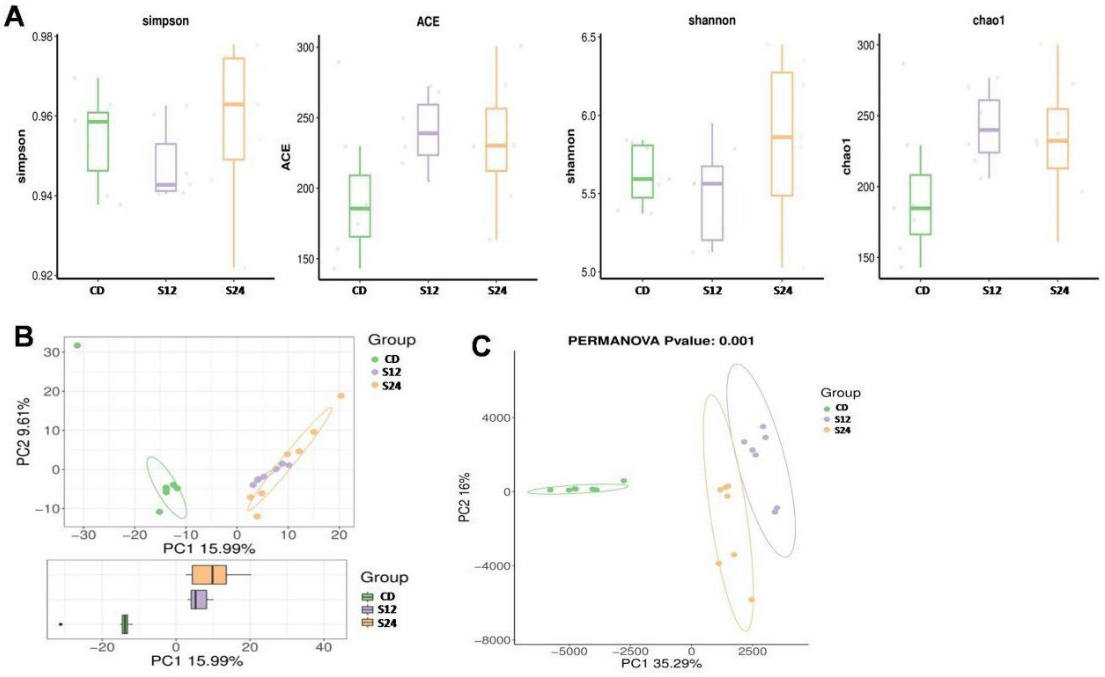

Figure S2. α-diversity and β-diversity analyses of the gut microbiota in NASH model. (A) Simpson index, ACE index, Shannon index, and Chao1 index on the ASVs level. (B)

PCA analysis and (C) PCoA analysis based on Bray-Curtis distance. Abbreviation: ASVs, amplicon sequence variants; PCA, principal component analysis; PCoA, principal coordinate analysis.

Supplementary Fig. 3

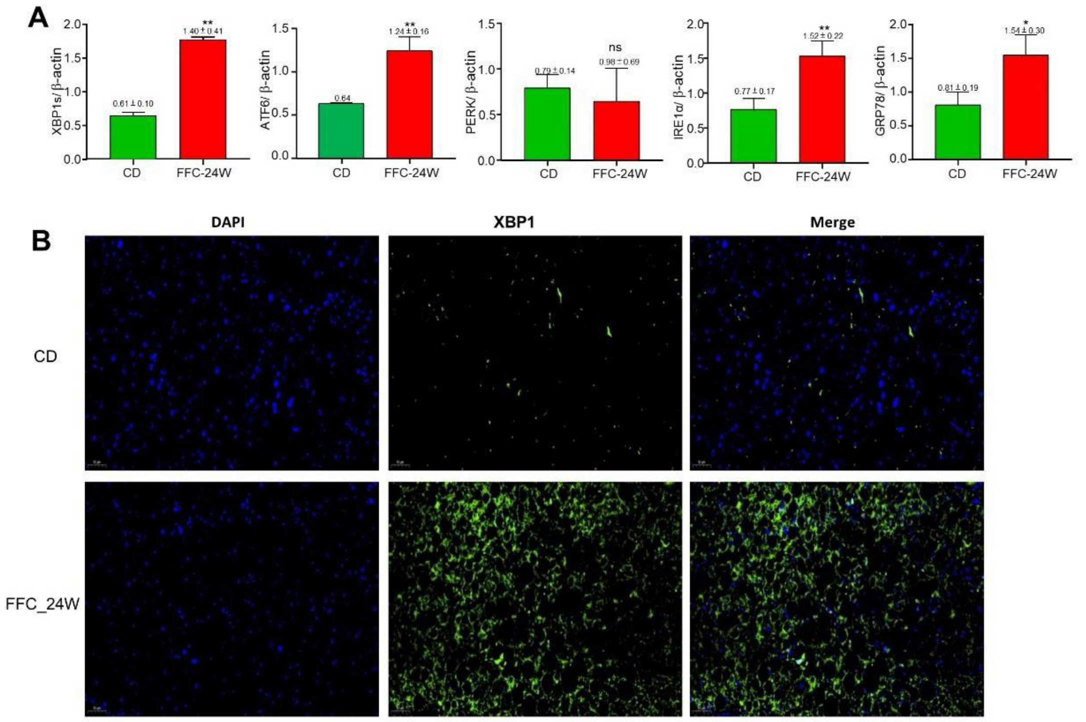

Figure S3. ER stress is activated in FFC diet induced NASH model. (A) Semi-quantitatively analyzed the band intensity of XBP1s, ATF6, IRE1α, GRP78 and PERK of Fig. 4A. (B) The expression of XBP1 in the liver tissues from mice fed with CD or FFC diet for 24 weeks was measured using immunofluorescence assay. Data are presented as the means ± SD (error bar) of at least three independent experiments. **P*< 0.05 and ***P*<0.01 as indicated.

Supplementary Fig. 4

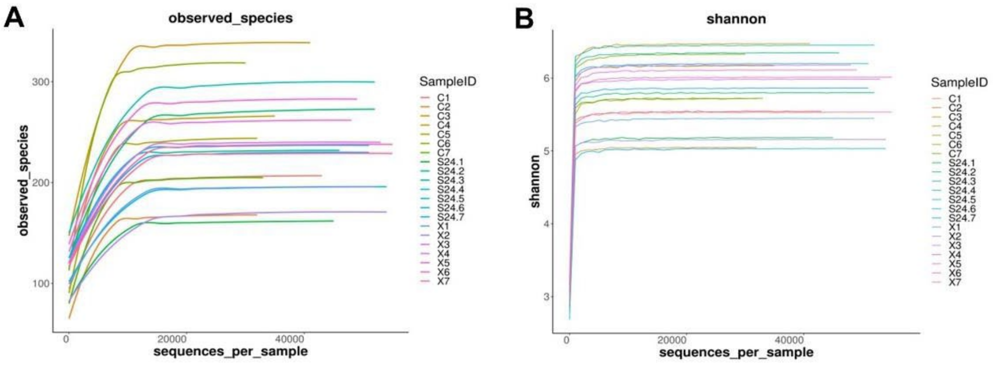

Figure S4. The rarefaction curve (A) and shannon curves (B) in FFC diet fed mice or mice treated with FT@*Xbp1*.

Supplementary Fig. 5

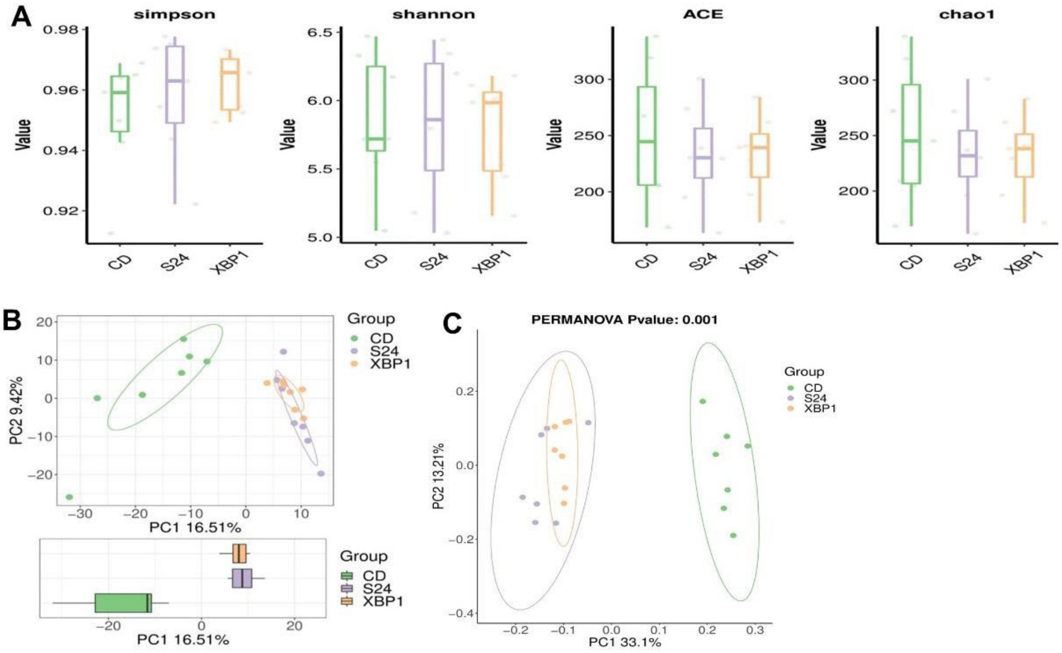

Figure S5. α-diversity and β-diversity analyses of the gut microbiota in mice treated with FT@*Xbp1*. (A) Simpson index, ACE index, Shannon index, and Chao1 index on the ASVs level. (B) PCA analysis and (C) PCoA analysis based on Bray-Curtis distance.

Supplementary Fig. 6

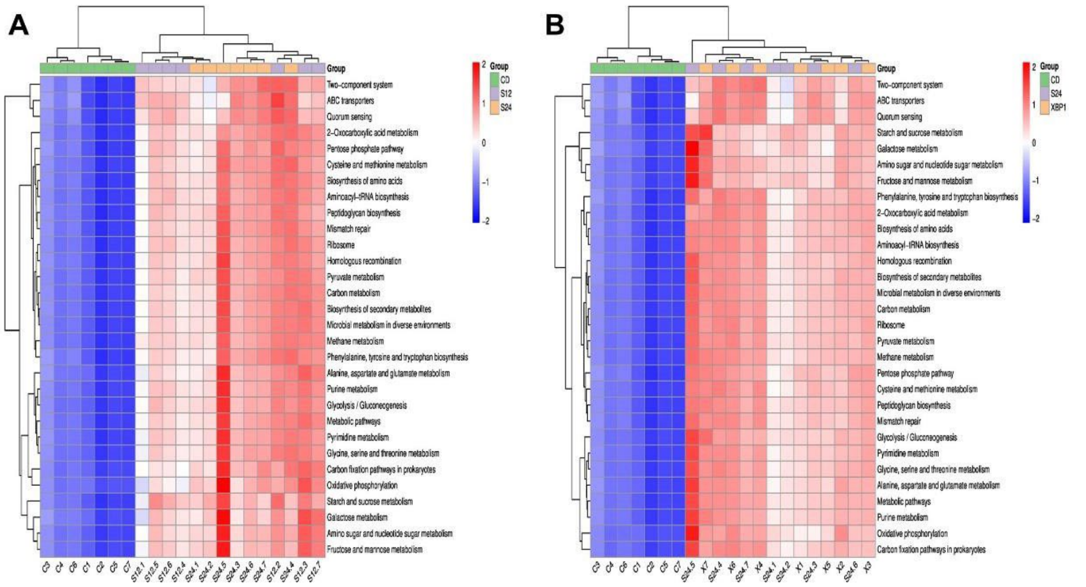

Figure S6. FT@*Xbp1* treatment prevented FFC diet-induced genes alteration from microbial community. (A) Heatmap recapitulates of Phylogenetic Investigation of Communities by Reconstruction of Unobserved States predicted by Kyoto Encyclopedia of Genes and Genomes pathways at the different stages of NASH. (B) Heatmap recapitulates of Phylogenetic Investigation of Communities by Reconstruction of Unobserved States predicted by Kyoto Encyclopedia of Genes and Genomes pathways in FFC diet fed mice or mice treated with FT@*Xbp1* (Kruskal–Wallis H-test, FDR < 0.1).
